# APP Causes Hyperexcitability in Fragile X Mice

**DOI:** 10.3389/fnmol.2016.00147

**Published:** 2016-12-15

**Authors:** Cara J. Westmark, Shih-Chieh Chuang, Seth A. Hays, Mikolaj J. Filon, Brian C. Ray, Pamela R. Westmark, Jay R. Gibson, Kimberly M. Huber, Robert K. S. Wong

**Affiliations:** ^1^Department of Neurology, University of Wisconsin-Madison, MadisonMadison, WI, USA; ^2^Department of Physiology and Pharmacology, State University of New York Downstate Medical CenterBrooklyn, NY, USA; ^3^Department of Neuroscience, University of Texas Southwestern Medical CenterDallas, TX, USA; ^4^Department of Medicine, University of Wisconsin-Madison, MadisonMadison, WI, USA

**Keywords:** amyloid-beta, amyloid-beta precursor protein, fragile X mental retardation protein, fragile X syndrome, hyperexcitability

## Abstract

Amyloid-beta protein precursor (APP) and metabolite levels are altered in fragile X syndrome (FXS) patients and in the mouse model of the disorder, *Fmr1*^*KO*^ mice. Normalization of APP levels in *Fmr1*^*KO*^ mice (*Fmr1*^*KO*^/*APP*^*HET*^ mice) rescues many disease phenotypes. Thus, APP is a potential biomarker as well as therapeutic target for FXS. Hyperexcitability is a key phenotype of FXS. Herein, we determine the effects of APP levels on hyperexcitability in *Fmr1*^*KO*^ brain slices. *Fmr1*^*KO*^/*APP*^*HET*^ slices exhibit complete rescue of UP states in a neocortical hyperexcitability model and reduced duration of ictal discharges in a CA3 hippocampal model. These data demonstrate that APP plays a pivotal role in maintaining an appropriate balance of excitation and inhibition (E/I) in neural circuits. A model is proposed whereby APP acts as a rheostat in a molecular circuit that modulates hyperexcitability through mGluR_5_ and FMRP. Both over- and under-expression of APP in the context of the *Fmr1*^*KO*^ increases seizure propensity suggesting that an APP rheostat maintains appropriate E/I levels but is overloaded by mGluR_5_-mediated excitation in the absence of FMRP. These findings are discussed in relation to novel treatment approaches to restore APP homeostasis in FXS.

## Introduction

Amyloid-beta protein precursor (APP) levels are dysregulated in numerous neurological disorders that are comorbid with a seizure phenotype including fragile X syndrome (FXS) (Westmark, 2013). FXS is a trinucleotide repeat disorder caused by a CGG repeat expansion at the 5′-end of the *FMR1* gene. Hypermethylation of the repeat expansion results in transcriptional silencing of the *FMR1* gene and loss of expression of fragile X mental retardation protein (FMRP) (Jin and Warren, 2000). FMRP is an RNA binding protein (RBP) that plays a pivotal role in synaptic function. It is one of numerous RBP that interact with *amyloid precursor protein* (*App*) mRNA to regulate post-transcriptional and/or translational events involved in the synthesis of APP (Westmark and Malter, 2012). Specifically, FMRP binds to a guanine-rich region in the coding region of *App* mRNA and regulates APP translation through a metabotropic glutamate receptor 5 (mGluR_5_)-dependent pathway (Westmark and Malter, 2007). We hypothesize that altered expression of APP in FXS contributes to disease severity. In support of this hypothesis, genetic knockout of one *App* allele in *Fmr1*^*KO*^ mice (*Fmr1*^*KO*^/*APP*^*HET*^ mice) reduces APP expression in the *Fmr1*^*KO*^ to wild type (WT) levels and rescues audiogenic-induced seizures (AGS), the percentage of mature spines, open field and marble burying behavioral phenotypes, and mGluR-LTD (Westmark et al., 2011). APP and metabolite levels are altered in *Fmr1*^*KO*^ mice and FXS patients (Sokol et al., 2006; Westmark et al., 2011; Erickson et al., 2014; Pasciuto et al., 2015; Ray et al., 2016). Thus, APP is a potential therapeutic target as well as blood-based biomarker for FXS (Berry-Kravis et al., 2013; Westmark et al., 2016), and it is of interest to determine the effect(s) of APP levels on additional disease phenotypes. Herein, we ascertain the effects of *App* knockdown on hyperexcitability in the *Fmr1*^*KO*^ mouse.

## Genetic reduction of *App* rescues hyperexcitability in *Fmr1^*KO*^* mice

The psychiatric phenotype of FXS includes hyperexcitability traits such as tactile defensiveness, attention deficits, hyperactivity, and hyperarousal to sensory stimulation (Tranfaglia, 2011). There is high comorbidity of epilepsy in FXS with electroencephalogram (EEG) patterns most often consisting of a centrotemporal spike pattern resembling Benign Focal Epilepsy of Childhood (BFEC) (Berry-Kravis, 2002). Hyperexcitability can be modeled in the *Fmr1*^*KO*^ mice both *in vivo* and *in vitro* (brain slices). *In vivo*, the *Fmr1*^*KO*^ mice are susceptible to AGS (Chen and Toth, 2001). In the AGS model, mice are exposed to 110 dB siren, which elicits out-of-control (wild) running and jumping followed by convulsive seizures and often death. There is substantial evidence that dysregulated APP expression alters seizure propensity. AGS are exacerbated by overexpression of APP in the *Fmr1*^*KO*^ mouse (FRAXAD mice) and partially rescued by reduced expression of APP in *Fmr1*^*KO*^*/APP*^*HET*^ mice (Westmark et al., 2010, 2011). Alzheimer's disease (Tg2576) and Down syndrome (Ts65Dn) mice, which overexpress human and mouse APP respectively, are highly susceptible to AGS (Westmark et al., 2010). Numerous mouse models that express altered APP or metabolite levels exhibit elevated rates of spontaneous or provoked seizures (Moechars et al., 1996; Steinbach et al., 1998; Del Vecchio et al., 2004; Lalonde et al., 2005; Palop et al., 2007; Kobayashi et al., 2008; Westmark et al., 2008; Minkeviciene et al., 2009; Ziyatdinova et al., 2011; Sanchez et al., 2012) while suppression of transgenic APP in Alzheimer's disease mice during postnatal development delays the onset of EEG abnormalities (Born et al., 2014).

**Figure 1:**
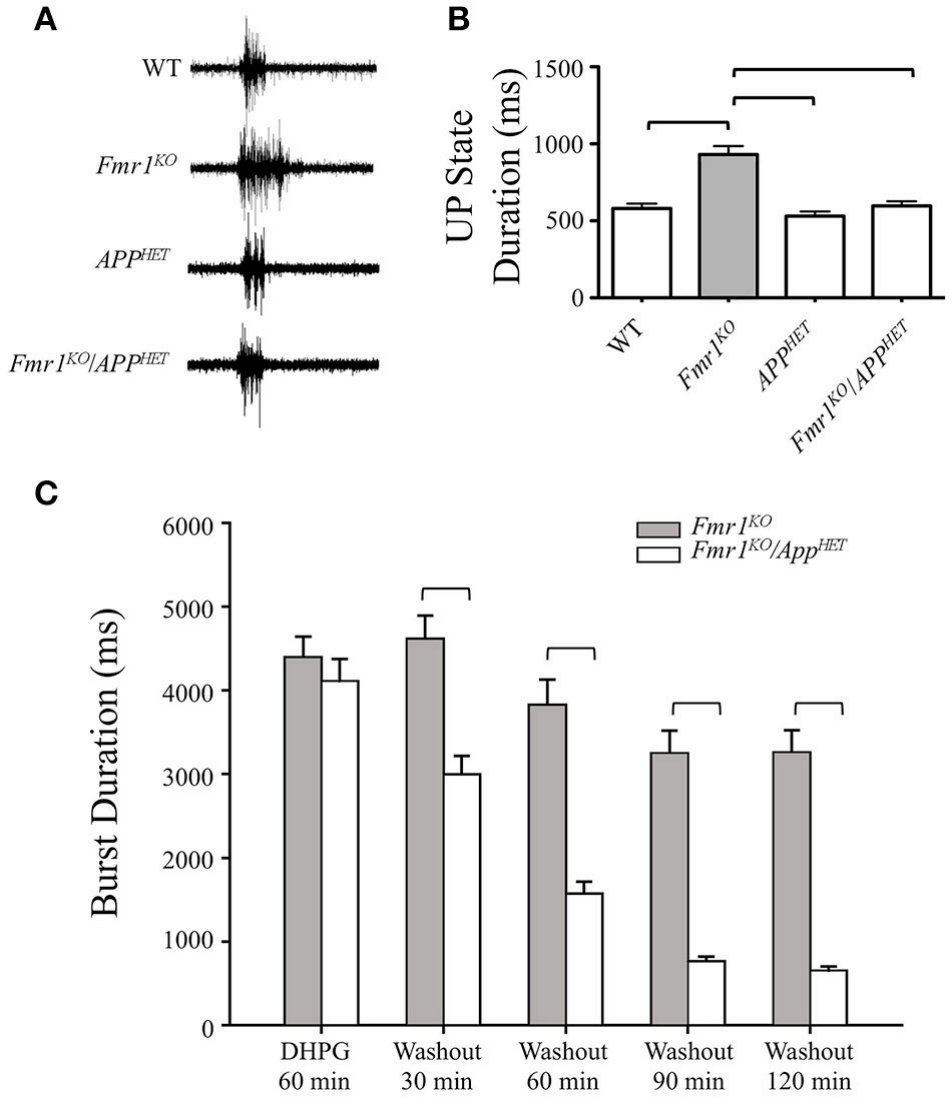
**Rescue of hyperexcitability in ***Fmr1***^***KO***^ mice by genetic manipulation of ***APP*****. Thalamocortical slices from WT (*n* = 17), *Fmr1*^*KO*^ (*n* = 22), *App*^*HET*^ (*n* = 13), and *Fmr1*^*KO*^*/App*^*HET*^ (*n* = 13) male mice were assessed for neocortical hyperexcitability. **(A)** Trace recordings and **(B)** histogram depicting a significant increase in UP state activity in *Fmr1*^*KO*^ slices compared to WT, which was completely rescued in the *Fmr1*^*KO*^*/App*^*HET*^. Error bars represent SEM. Horizontal bars denote statistically different levels by one-way ANOVA and Bonferroni's multiple comparison test (*P* < 0.0001). DHPG-induced prolonged epileptiform discharges were assessed in hippocampal slices from *Fmr1*^*KO*^ and *Fmr1*^*KO*^*/App*^*HET*^ male mice (*n* = 6 mice per cohort). The recordings were continuous for 3 or more hours in a single slice per animal. **(C)** Summary frequency histogram of synchronized epileptiform discharges from *Fmr1*^*KO*^ and *Fmr1*^*KO*^*/App*^*HET*^ slices in the presence of DHPG (60 min) and after DHPG washout at the indicated times up to 2 h. The mean durations of epileptiform discharges in *Fmr1*^*KO*^*/App*^*HET*^ slices at 30, 60, 90, and 120 min after DHPG washout are significantly shorter than those in *Fmr1*^*KO*^ for all times tested (*P* < 0.001).

In brain slices, hyperexcitability can be measured by recording UP states and epileptiform discharges. UP states are short periods of local network activity that generate a steady-state level of depolarization and synchronous firing among groups of neighboring neurons (Gibson et al., 2008). *Fmr1*^*KO*^ mice exhibit an increased duration of the UP state, consistent with network hyperexcitability (Gibson et al., 2008; Goncalves et al., 2013). Specifically, spontaneously occurring UP states are 38-67% longer in *Fmr1*^*KO*^ than in WT slices (Hays et al., 2011). Deletion of *Fmr1* selectively in excitatory neurons mimics the prolonged UP states whereas knockdown of mGluR_5_ rescues the hyperexcitability in the *Fmr1*^*KO*^ with no effect in WT (Hays et al., 2011). To determine if hyperexcitability was rescued in *Fmr1*^*KO*^ mice by knockdown of *App*, we recorded UP states in *Fmr1*^*KO*^*/App*^*HET*^ mice and littermate controls per previously described methods (Gibson et al., 2008). Briefly, *Fmr1*^*HET*^*/App*^*HET*^ females were bred with *App*^*HET*^ males to generate WT, *Fmr1*^*KO*^, *App*^*HET*^ and *Fmr1*^*KO*^*/App*^*HET*^ male littermates. Thalamocortical slices (400 μm) from postnatal day 24–28 (P24-P28) males were transected parallel to the pia mater to remove the thalamus and midbrain, and spontaneously generated UP states were recorded in layer 4 of the somatosensory cortex. The increased duration of the UP states observed in the *Fmr1*^*KO*^ was completely rescued in *Fmr1*^*KO*^/*APP*^*HET*^ mice (Figures 1A,B) where UP state duration decreased from 931 ± 55 milliseconds (ms) in *Fmr1*^*KO*^ to 597 ± 30 ms in *Fmr1*^*KO*^/*APP*^*HET*^, (*p* < 0.001). UP state duration was not significantly different between *APP*^*HET*^ and WT slices suggesting that rescue was not a consequence of a general reduction in excitability due to lower APP levels.

Hyperexcitability can also be evaluated in slices of the CA3 region of the hippocampus in *Fmr1*^*KO*^ mice. Prolonged epileptic bursts can be induced by group 1 mGluR agonists in both WT and *Fmr1*^*KO*^ mice and with a GABAergic antagonist only in *Fmr1*^*KO*^ (Chuang et al., 2005; Zhong et al., 2009). In WT slices, DHPG elicits short (~500 ms) synchronized discharges that gradually extend to reach an average duration of 4.4 ± 0.14 s at 60 min; and in untreated *Fmr1*^*KO*^ slices, bicuculline elicits short <1 ms synchronized discharges that progressively increase in duration over 60 min (average duration 2.3 ïĆś 0.13 s) (Osterweil et al., 2013). These prolonged epileptiform discharges resemble the ictal discharges observed in the CA3 region in epilepsy (Merlin and Wong, 1997; Wong et al., 2004). The number and duration of ictal-like discharges were assessed by intracellular CA3 recordings in juvenile *Fmr1*^*KO*^ and *Fmr1*^*KO*^*/App*^*HET*^ slices in the presence of DHPG (60 min) and after DHPG washout for up to 2 h as previously described (Chuang et al., 2005) (Figure 1C, Supplementary Figure 1). In the presence of DHPG, a distinct population of ictal-like discharges (burst duration > 1500 ms) occurred in both *Fmr1*^*KO*^ and *Fmr1*^*KO*^*/App*^*HET*^ slices. After DHPG washout, the ictal-like discharges remained distinct for the duration of the recording (up to 2 h post-DHPG washout) in the *Fmr1*^*KO*^, but not in the *Fmr1*^*KO*^*/App*^*HET*^ slices. Thus, a major difference between *Fmr1*^*KO*^ and *Fmr1*^*KO*^*/App*^*HET*^ slices is that while ictal-like discharges were transiently expressed in both genotypes, they were not maintained in the *Fmr1*^*KO*^*/App*^*HET*^ upon termination of receptor stimulation. The seizure activity modeled in the hippocampal slice paradigm is congruent with the AGS phenotype observed in *Fmr1*^*KO*^*/App*^*HET*^ mice where wild running and seizures are attenuated but not completely rescued to WT levels (Westmark et al., 2011). The two critical components of plasticity include the initiating factors required for induction of the modification and the downstream effectors that maintain expression of the enhanced response (Bianchi et al., 2012). Our data suggest that genetic reduction of *App* in the *Fmr1*^*KO*^ background does not prevent the induction of seizure activity, but can attenuate progression; thus, APP appears to be a downstream effector that maintains hyperexcitability in the context of the *Fmr1*^*KO*^.

The complete rescue of hyperexcitability in the neocortex compared to the partial rescue in the hippocampus in the *Fmr1*^*KO*^/*App*^*HET*^ mice is in accord with studies in immature mice demonstrating that the hippocampus has a lower seizure threshold compared to neocortex (Abdelmalik et al., 2005). This could be due differential expression and/or activity of group 1 mGluRs (mGluR_1_ and mGluR_5_) in the respective neurons under study. In fast spiking inhibitory neurons (neocortical slice model), mGluR_1_ is more highly expressed than mGluR_5_ (Sun et al., 2009); however reduced expression of mGluR_5_ or APP in the *Fmr1*^*KO*^ completely rescues neocortical hyperexcitability whereas UP states are still longer in the *Fmr1*^*KO*^ after treatment with the mGluR_1_ inhibitor LY367385 (Hays et al., 2011). These data suggest that mGluR_5_ is the critical group 1 mGluR that modulates *Fmr1*-dependent hyperexcitability in the neocortex. Alternatively, in CA3 hippocampal neurons, both group 1 mGluR subtypes are involved in the induction and maintenance of mGluR-mediated bursts, but mGluR_5_ plays a greater role in the induction and mGluR_1_ in the maintenance of the prolonged epileptic bursts (Merlin, 2002). As burst duration but not induction are rescued in the *Fmr1*^*KO*^*/APP*^*HET*^, these data suggest that the hyperexcitability elicited by elevated APP expression in the *Fmr1*^*KO*^ CA3 region is dependent on mGluR_1_.

Synaptic dysfunction occurs when the appropriate balance of excitation and inhibition (E/I) in neural circuits is not maintained (Gatto and Broadie, 2010). The absence of FMRP during postnatal development results in an E/I imbalance dominated by excitation. Our results demonstrate that E/I balance is predominantly restored when APP expression is reduced to WT levels in the *Fmr1*^*KO*^. Thus, APP plays a critical role in modulating excitability. The other half of E/I balance is the inhibitory feedback on circuits. FMRP normally binds to multiple GABA_*A*_R mRNAs, and their expression is decreased in juvenile *Fmr1*^*KO*^ (Braat et al., 2015) resulting in delay of the developmental GABA switch in *Fmr1*^*KO*^ (He et al., 2014). Selective deletion of *Fmr1* in inhibitory neurons has no effect on prolonged UP states suggesting that impaired GABA_*A*_R signaling in FXS does not account for increased hyperexcitability in the neocortex (Hays et al., 2011). Conversely, a competitive antagonist of GABA_*A*_R, bicuculline, elicits epileptiform discharges in the CA3 region of the hippocampus (Osterweil et al., 2013). These findings suggest that inhibitory feedback is differentially regulated in the neocortex and hippocampus in *Fmr1*^*KO*^. Overall, the neocortical hyperexcitability and hippocampal epileptiform discharge slice models share the features of prolonged activity states and dependence on mGluR_5_, FMRP, and APP, but differ in induction mode (neocortical slices exhibit baseline excitation vs. hippocampal slices require pharmacological stimulation), inhibitory feedback (hippocampal slices are dependent of GABA_*A*_R), and protein synthesis requirements (CA3 bursts require extracellular signal-regulated kinase (ERK)1/2 activation and new protein synthesis) (Zhao et al., 2004; Chuang et al., 2005; Hays et al., 2011).

## A model for an APP-induced short circuit in fragile X

Regarding possible mechanisms for APP-mediated hyperexcitability, (Westmark, 2013) APP or a metabolite could interfere with cell surface receptor activation. For example, Aβ oligomers cause redistribution of mGluR_5_to synapses (Renner et al., 2010) and trigger multiple distinct signaling events through mGluR_5_/prion protein complexes (Um et al., 2013; Hu et al., 2014; Haas and Strittmatter, 2016). In neurons that overexpress APP, Aβ depresses excitatory synaptic transmission (Kamenetz et al., 2003). In *Fmr1*^*KO*^ mice, Aβ levels are elevated in older mice but reduced in juvenile mice compared to WT controls (Westmark et al., 2011; Pasciuto et al., 2015). Thus, increased α-secretase and/or decreased BACE1 processing during postnatal development could result in decreased Aβ levels and increased synaptic transmission (Jin and Warren, 2000) Altered APP expression could affect scaffolding protein interactions at the postsynaptic density. For example, APP co-immunoprecipitates with Homer2 and Homer3 (Parisiadou et al., 2008). These scaffolding proteins inhibit APP processing, reduce cell surface APP expression, and prevent maturation of BACE1 (Parisiadou et al., 2008). Uncoupled Homer1-mGluR_5_ interactions underlie *Fmr1*^*KO*^ phenotypes, and genetic deletion of Homer1a rescues prolonged UP states in *Fmr1*^*KO*^ mice similar to the complete rescue observed herein in the *Fmr1*^*KO*^*/APP*^*HET*^ mice (Ronesi et al., 2012). APP does not co-immunoprecipitate with Homer1 (Parisiadou et al., 2008); however, Aβ induces disassembly of Homer1b and Shank1 clusters (Roselli et al., 2009). (Westmark and Malter, 2012) APP or metabolites could alter the activity of intracellular signaling pathways such as ERK and mTOR (Young et al., 2009; Ma et al., 2010; Caccamo et al., 2011; Chasseigneaux et al., 2011; Pasciuto et al., 2015). Both of these pathways play pivotal roles in FXS pathology (Osterweil et al., 2010; Hoeffer et al., 2012). And Westmark and Malter (2007) APP metabolites could function in feedback loops to regulate the aforementioned pathways or even the transcription of the APP and APP processing enzymes. Aβ binds to the promoter regions of the APP and BACE1 genes and may function as a transcription factor to regulate its own production and/or processing (Bailey et al., 2011). Thus, there are numerous molecular junctures where altered expression of APP or metabolites could interfere with synaptic function and lead to a hyperexcitable circuit.

**Figure 2:**
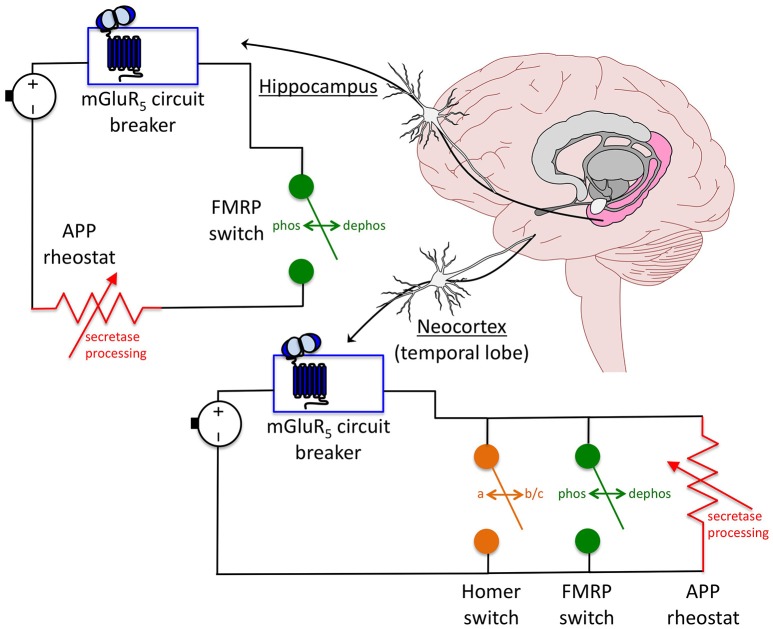
**Model for an APP-induced short circuit in FXS**. APP acts as a rheostat (i.e., variable resistor, dimmer switch) in a circuit where mGluR_5_ inhibitors are a circuit breaker and FMRP is an automatic transfer switch that regulate neuronal excitability. The FMRP switch is dependent on a rapid dephosphorylation reaction in response to mGluR_5_ activation. In the absence of FMRP, the circuit is constitutively on. In the presence of mGluR_5_ inhibitors, the circuit is shut down. The downstream APP circuitry appears to be wired differently dependent on brain region. In the neocortex, knockdown of individual proteins including mGluR_5_, Homer and APP completely rescues excitability levels suggesting that these components are arranged in a parallel circuit whereby there is more than one continuous signaling pathway between mGluR_5_ activation and excitability output. Rescue of any one of the parallel components is sufficient to restore synaptic homeostasis. In the hippocampus, ictal burst duration, but not induction, is rescued in *Fmr1*^*KO*^*/App*^*HET*^ slices in response to DHPG treatment. This incomplete rescue suggests that APP and FMRP are wired in series downstream of mGluR_5_, and that the APP rheostat is overloaded in the absence of FMRP resulting in a short circuit. This model has important implications for therapeutic development and suggests that APP should be considered as a drug target for FXS as part of a multi-drug therapeutic strategy.

Overall, these data suggest a model whereby mGluR_5_ inhibitors act as a circuit breaker, FMRP as an automatic transfer switch and APP as a rheostat in a circuit that controls hyperexcitability (Figure 2). *The mGluR*_5_ *circuit breaker*: Genetic reduction of mGluR_5_ in the *Fmr1*^*KO*^ mouse rescues plasticity (ocular dominance plasticity, neocortical hyperexcitability), dendritic spines (density on cortical pyramidal neurons), protein synthesis, behavior (inhibitory avoidance extinction), and AGS (Dolen et al., 2007; Hays et al., 2011). Pharmaceutical inhibition of mGluR_5_ likewise rescues numerous *Fmr1*^*KO*^ phenotypes (Michalon et al., 2012, 2014). Thus, inhibiting mGluR_5_ appears to break a circuit that mediates hyperexcitability in the *Fmr1*^*KO*^ mouse. *The FMRP automatic transfer switch*: mGluR_5_ activation causes a rapid dephosphorylation of FMRP, which permits protein synthesis (Ceman et al., 2003; Narayanan et al., 2007), as well as a biphasic change in FMRP levels (initial decrease followed by increase) (Zhao et al., 2011). Thus, FMRP appears to function as an automatic transfer switch downstream of mGluR_5_ to control protein synthesis in response to receptor activation. In FXS models, loss of the FMRP switch that modulates mGluR_5_ signaling permits a constitutively-on circuit. *The APP rheostat*: Born and colleagues demonstrated that juvenile overexpression of APP contributes to sharp wave EEG discharges in APP transgenic mice, and proposed that APP expression functions as a rheostat that regulates synaptic balance in the brain (Born et al., 2014). We have observed that both over- and under-expression of APP increases seizure propensity in juvenile *Fmr1*^*KO*^ mice suggesting that tight regulation of this protein may be necessary to mitigate hyperexcitability in FXS (Westmark et al., 2010, 2011). Genetic reduction of APP in *Fmr1*^*KO*^ mice rescues plasticity (mGluR-LTD, neocortical hyperexcitability, epileptiform discharge duration but not induction), dendritic spines (percent mature spines but not dendritic spine length), protein synthesis, behavior (open field, marble burying), and AGS (Westmark et al., 2011; Pasciuto et al., 2015). The partial rescue of dendritic spines and epileptiform discharges in the *Fmr1*^*KO*^*/APP*^*HET*^ suggests that APP is necessary but not sufficient to maintain synaptic homeostasis. Thus, in the context of WT mice, an APP variable resistor is capable of maintaining an appropriate E/I balance, but in *Fmr1*^*KO*^ and some APP transgenic mice, excess APP appears to cause a short circuit through overload of the APP rheostat resulting in hyperexcitability. Likewise, complete loss of APP would bypass the APP rheostat. *Fmr1*^*KO*^*/App*^*KO*^ mice exhibit an extremely strong AGS phenotype (97%, *n* = 36 mice) (Westmark et al., 2013a), which is not observed in *App*^*KO*^ mice (11%, *n* = 36 mice). These data suggest that exacerbated hyperexcitability is a result of the combined loss of both FMRP and APP.

APP and metabolites play key roles in regulating synaptic activity with both Aβ and sAPPα implicated in positive feedback loops that facilitate mGluR_5_ signaling (Casley et al., 2009; Renner et al., 2010; Ferreira and Klein, 2011; Westmark et al., 2011, 2013b; Pasciuto et al., 2015). Thus, the APP rheostat may provide a graded response to mGluR_5_ activation through feedback loops involving amyloidogenic and non-amyloidogenic secretase processing. We found that AGS are attenuated in *Fmr1*^*KO*^ mice with BACE1 inhibitor treatment (Supplementary Figure 2). Prox and colleagues found that seizures are increased in the ADAM10 conditional knockout mouse (loss of α-secretase processing) (Prox et al., 2013). The effect of sAPPα overexpression on hyperexcitability, which could be studied in TgAPPα (Bailey et al., 2013) and TgAPPα/*Fmr1*^*KO*^ mice, remains to be determined. Thus, multiple APP fragments may play roles in hyperexcitability and seizure susceptibility. A caveat to this model is that over-expression of APP alone is not sufficient to increase seizure propensity in either WT or *Fmr1*^*KO*^ mice. We tested seizures in two alternative Alzheimer's disease mouse models, R1.40 and J20, which exhibit elevated APP expression (Lamb et al., 1997; Mucke et al., 2000). Neither strain exhibited a strong AGS phenotype (Supplementary Figures 3, 4) in contrast to Tg2576 and Ts65Dn (Westmark et al., 2010). A genetic cross of J20 with *Fmr1*^*KO*^ mice that produced mice over-expressing human APP in the context of the *Fmr1*^*KO*^ background did not result in exacerbated AGS rates in comparison to *Fmr1*^*KO*^ unlike the FRAXAD mice (cross of Tg2576 with *Fmr1*^*KO*^) (Westmark et al., 2010). The inclusion of flanking sequences in the transgenic constructs used for the R1.40 and J20 mice are expected to affect posttranscriptional regulation of the *APP* gene, which could alter the temporal and spatial expression of APP and metabolites and thus their contribution to seizure threshold. Of note, *Fmr1*^*HET*^/J20 female mice exhibited a 50% wild running rate, which was significantly higher than WT, *Fmr1*^*HET*^ and J20 controls, supporting the assertion that APP works in synergy with FMRP to regulate hyperexcitability (Supplementary Figure 4). This synergistic effect is also observed in mGluR-LTD studies where loss of FMRP and APP modulate synaptic transmission in opposite directions (Westmark et al., 2011). The *Fmr1*^*KO*^/*APP*^*HET*^ mice used herein were a constitutive *App* knockdown. It remains to be determined how conditional knockdown of *App* during development affects *Fmr1* phenotypes.

## Relevance to therapeutic development

All major *Fmr1*^*KO*^ phenotypes can be corrected by inhibition or knockdown of mGluR_5_ in mice; however, neural circuitry is likely more complicated in humans and it may be necessary to employ pharmaceutical cocktails for disease treatment. Drugs under study for FXS such as acamprosate, AFQ056, donepezil, ganaxolone, lithium, lovastatin, memantine, minocycline and sertraline exhibit on- and/or off-site effects that are expected to modulate APP, Aβ, BACE1, and/or ADAM10 (Westmark et al., 2013b). Targeting APP and metabolites in FXS may allow fine tuning of excitability levels as part of a multi-drug therapeutic approach. Both amyloidogenic and non-amyloidogenic therapies have been proposed to treat FXS (Westmark et al., 2013b; Pasciuto et al., 2015). Both amyloidogenic (Aβ_1−42_) and non-amyloidogenic (sAPPα) metabolites of APP stimulate phosphorylation of ERK and modulate synthesis of multiple synaptic proteins predicted to be regulated through mGluR_5_/FMRP and to contribute to altered synaptic plasticity (Westmark et al., 2011; Pasciuto et al., 2015). Thus, it may be necessary to simultaneously modulate both α- and β-secretase processing to attain homeostatic levels of APP metabolites and rescue hyperexcitability in FXS.

## Author contributions

CW, JG, KH, RW conceived and designed the experiments. CW, SC, SH, MF, BR, PW acquired data. CW, SC, SH, JG, KH, RW interpreted data. CW drafted the manuscript.

## Funding

This work was funded by FRAXA Research Foundation and NIH R21AG044714 (CW), and NIH 1R01HD056370 (JG).

### Conflict of interest statement

The authors declare that the research was conducted in the absence of any commercial or financial relationships that could be construed as a potential conflict of interest.
