# Supplementary material for: APP Causes Hyperexcitability in Fragile X Mice
Source: Front Mol Neurosci. 2016 Dec 15;9:147. doi: 10.3389/fnmol.2016.00147 (PMC5156834; doi:10.3389/fnmol.2016.00147)
Supplement: Supplementary file 1 [file Image1.PDF]

Supplementary Material:
APP Causes Hyperexcitability in Fragile X Mice

Cara J. Westmark[1*], Shih-Chieh Chuang[2+], Seth A. Hays[3+], Mikolaj J. Filon[1], Brian C. Ray[1], Pamela R. Westmark[4], Jay R. Gibson[3], Kimberly M. Huber[3], Robert K. S. Wong[2]

*Correspondence:
Author Name: Cara Westmark
Email: westmark@facstaff.wisc.edu

Supplementary Figure 1

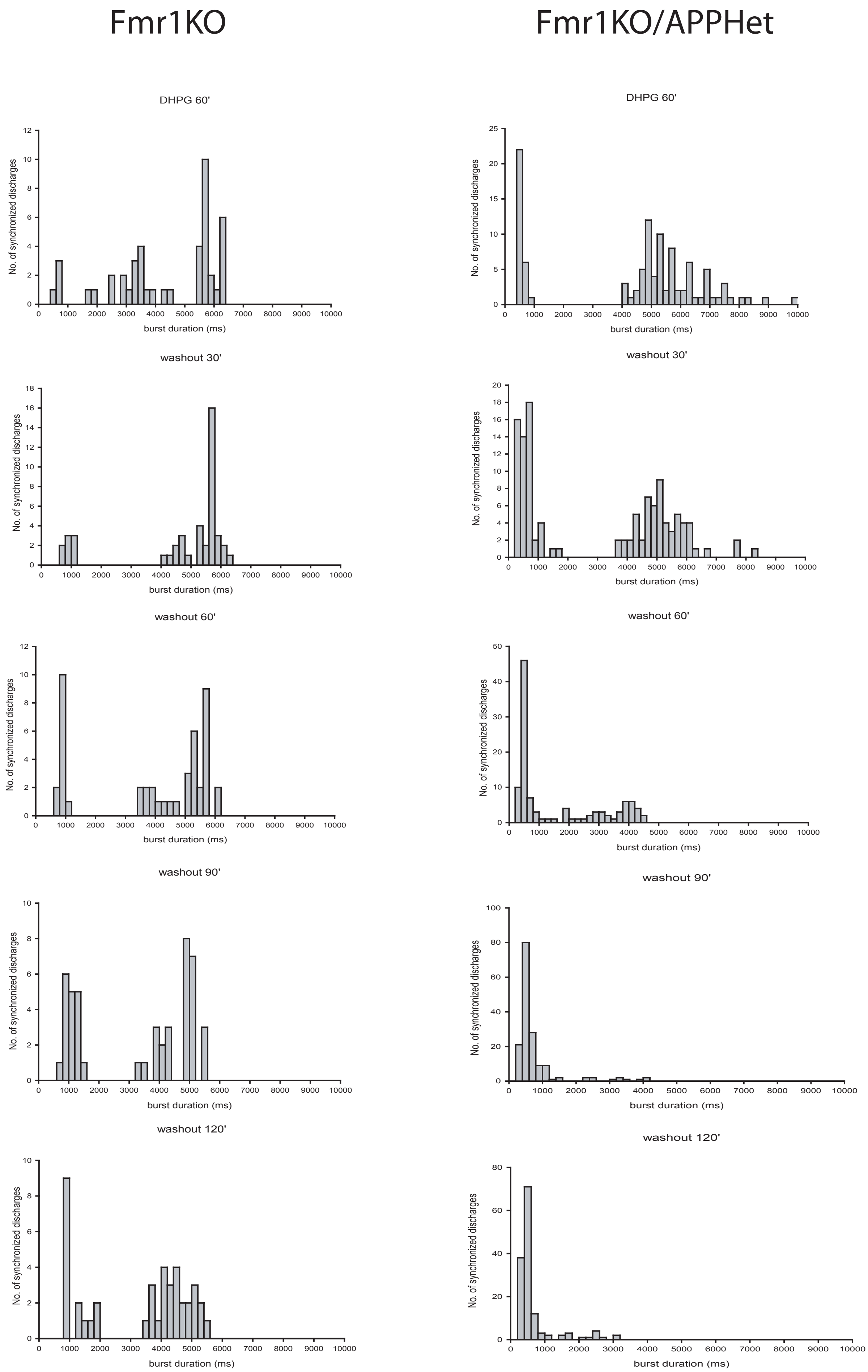

Supplementary Figure 1

Legend: DHPG-induced prolonged epileptiform discharges were assessed in hippocampal slices from *Fmr1*^{KO} and *Fmr1*^{KO}/*App*^{HET} male mice (n=6 mice per cohort). The recordings were continuous for 3 or more hours in a single slice per animal. Histograms of the mean duration of synchronized discharges in *Fmr1*^{KO} and *Fmr1*^{KO}/*App*^{HET} slices in the presence of DHPG (60 min) and after DHPG washout at the indicated times up to 2 hr. The mean duration of epileptiform discharge (inclusive of all short and prolonged discharges) for each group at DHPG 60 min is not statistically different (*Fmr1*^{KO}, 4393 ± 273 ms, n=45, and *Fmr1*^{KO}/*App*^{HET}, 4111 ± 265 ms, n=95; two-way ANOVA followed by Newman-Keuls post-hoc test: P=0.336). The mean durations of epileptiform discharges in *Fmr1*^{KO}/*App*^{HET} slices at 30, 60, 90 and 120 min after DHPG washout are significantly shorter than those in *Fmr1*^{KO} for all times tested (P<0.001).

Supplementary Figure 2

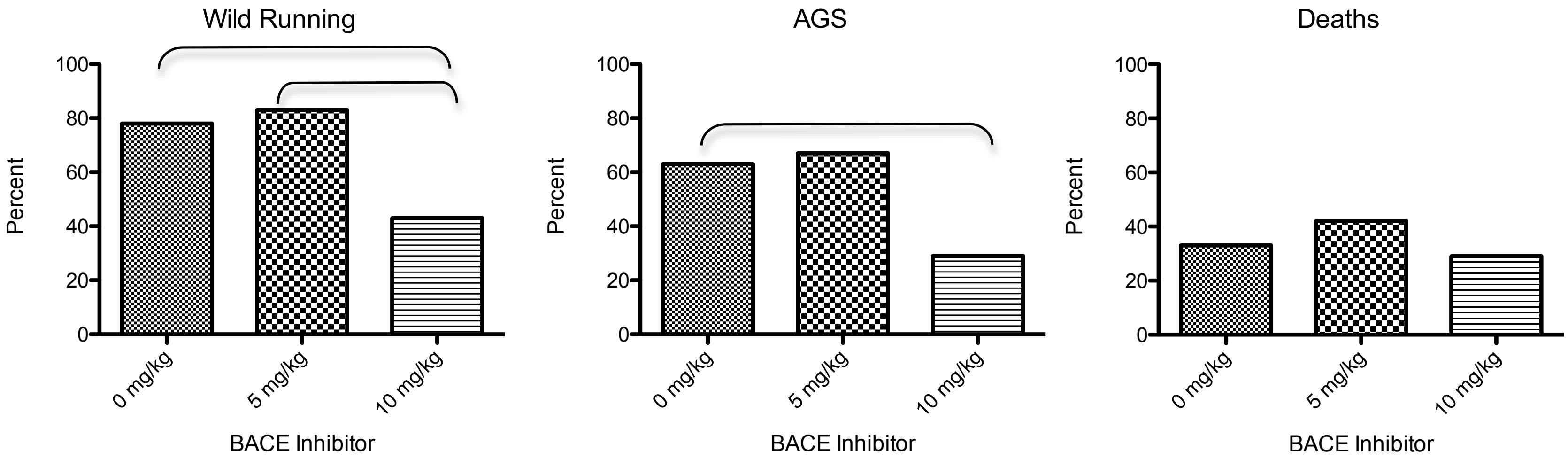

Legend: BACE inhibitor reduces AGS in *Fmr1*^{KO} mice. *Fmr1*^{KO} mice (postnatal day 21) were treated with vehicle (4% DMSO in DPBS) (n=27 mice) versus β-secretase inhibitor IV (CalBiochem catalog #565788) at 5 mg/kg (n=12 mice) and 10 mg/kg (n=14 mice) by I.P. injection 30 min before seizure testing. Percent WR, AGS and deaths were scored and plotted versus treatment condition. Bars represent statistically significant differences by Fisher exact test.

Supplementary Figure 3

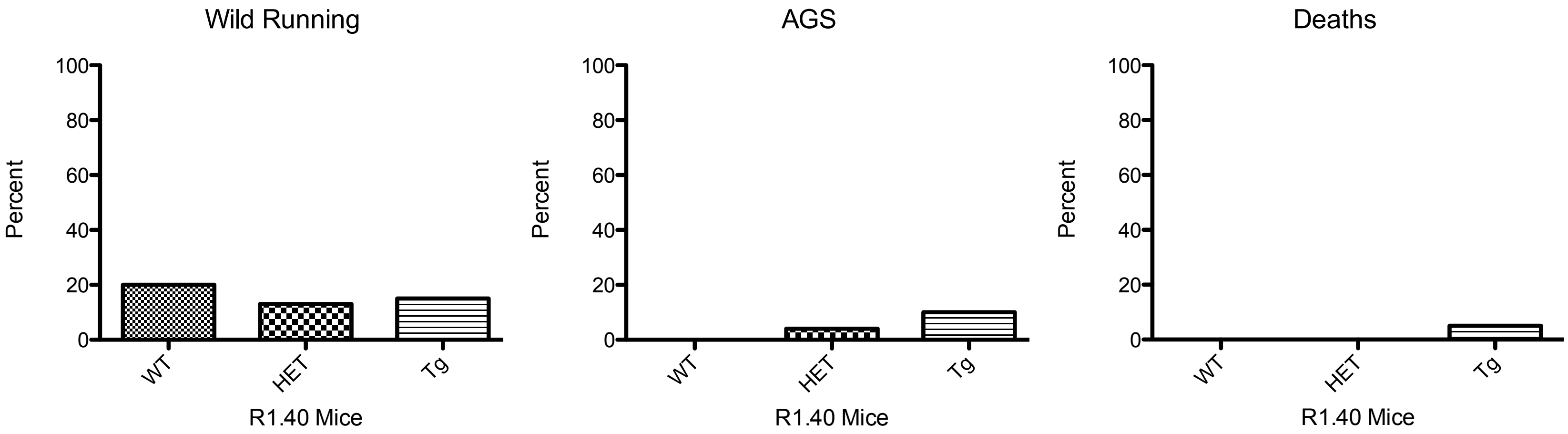

Legend: R1.40 mice do not exhibit a strong AGS phenotype. Juvenile littermates mice (postnatal day 21) were tested in the AGS paradigm: WT female (n=5), R1.40 HET (single copy transgene) (n=24), and R1.40 Tg (2 copies transgene) (n=20). Percent WR, AGS and deaths were scored and plotted versus genotype.

Supplementary Figure 4

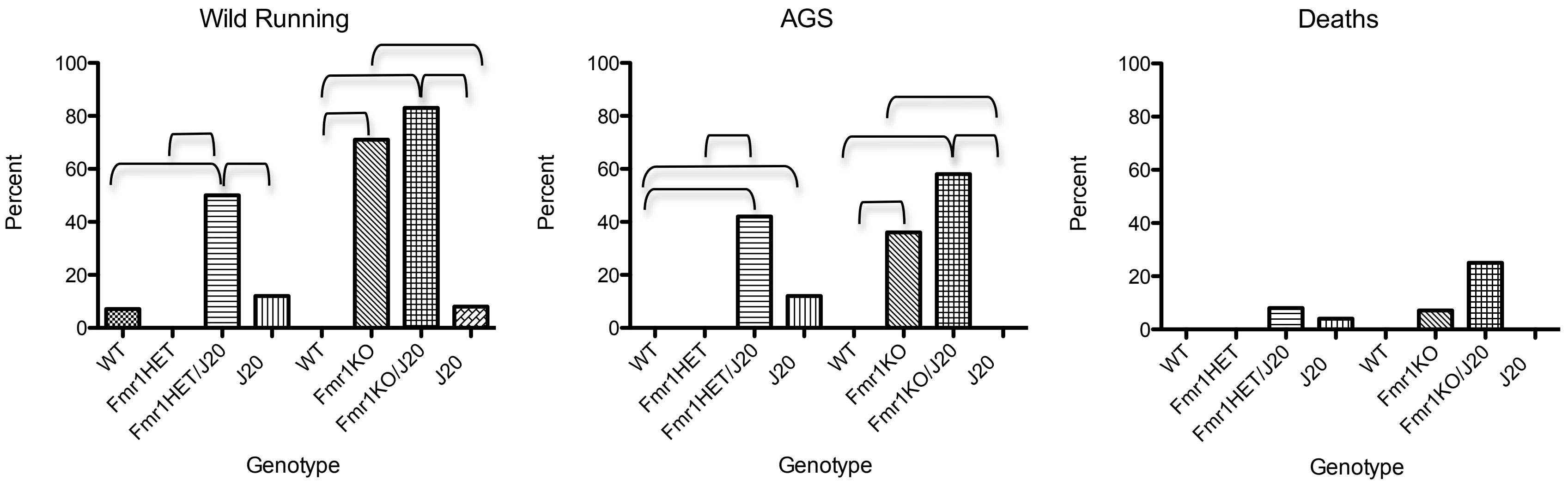

Legend: J20 mice do not exhibit a strong AGS phenotype. Juvenile mice (postnatal day 21) were tested in the AGS paradigm: WT female (n=45), *Fmr1*^{HET} female (n=11), *Fmr1*^{HET}/J20 female (n=12), J20 female (n=25), WT male (n=20), *Fmr1*^{KO} male (n=14), *Fmr1*^{KO}/J20 male (n=12), and J20 male (n=12). Percent WR, AGS and deaths were scored and plotted versus genotype. Bars denote statistically significant differences between genotypes by Fisher exact test. Of note, *Fmr1*^{HET}/J20 females exhibit 42% AGS compared to 0% AGS in *Fmr1*^{HET} suggesting that APP and FMRP act in synergy.
